# Longitudinal monitoring of amyotrophic lateral sclerosis by diffusion tensor imaging: Power calculations for group studies

**DOI:** 10.3389/fnins.2022.929151

**Published:** 2022-08-10

**Authors:** Anna Behler, Dorothée Lulé, Albert C. Ludolph, Jan Kassubek, Hans-Peter Müller

**Affiliations:** ^1^Department of Neurology, University of Ulm, Ulm, Germany; ^2^Deutsches Zentrum für Neurodegenerative Erkrankungen (DZNE), Ulm, Germany

**Keywords:** Amyotrophic Lateral Sclerosis, Diffusion Tensor Imaging, longitudinal design, statistical power, study optimization

## Abstract

**Introduction:**

Diffusion tensor imaging (DTI) can be used to map disease progression in amyotrophic lateral sclerosis (ALS) and therefore is a promising candidate for a biomarker in ALS. To this end, longitudinal study protocols need to be optimized and validated regarding group sizes and time intervals between visits. The objective of this study was to assess the influences of sample size, the schedule of follow-up measurements, and measurement uncertainties on the statistical power to optimize longitudinal DTI study protocols in ALS.

**Patients and methods:**

To estimate the measurement uncertainty of a tract-of–interest-based DTI approach, longitudinal test-retest measurements were applied first to a normal data set. Then, DTI data sets of 80 patients with ALS and 50 healthy participants were analyzed in the simulation of longitudinal trajectories, that is, longitudinal fractional anisotropy (FA) values for follow-up sessions were simulated for synthetic patient and control groups with different rates of FA decrease in the corticospinal tract. Monte Carlo simulations of synthetic longitudinal study groups were used to estimate the statistical power and thus the potentially needed sample sizes for a various number of scans at one visit, different time intervals between baseline and follow-up measurements, and measurement uncertainties.

**Results:**

From the simulation for different longitudinal FA decrease rates, it was found that two scans per session increased the statistical power in the investigated settings unless sample sizes were sufficiently large and time intervals were appropriately long. The positive effect of a second scan per session on the statistical power was particularly pronounced for FA values with high measurement uncertainty, for which the third scan per session increased the statistical power even further.

**Conclusion:**

With more than one scan per session, the statistical power of longitudinal DTI studies can be increased in patients with ALS. Consequently, sufficient statistical power can be achieved even with limited sample sizes. An improved longitudinal DTI study protocol contributes to the detection of small changes in diffusion metrics and thereby supports DTI as an applicable and reliable non-invasive biomarker in ALS.

## Introduction

During the last decade, magnetic resonance imaging (MRI)-based parameters have gained increasing interest as a progression marker in neurodegenerative diseases (Agosta et al., 2015). Amyotrophic Lateral Sclerosis (ALS) is characterized by progressive motor neuron degeneration of both the upper motor neurons of the cerebral cortex and the lower motor neurons in the brainstem and spinal cord, leading to progressive immobility and breathing difficulties, and eventually died after an average of 3 years (van Es et al., 2017). In clinical trials, objective biomarkers, for example, based upon neuroimaging are needed to monitor the progression of the disease and thus improve the chances of identifying effective treatments for ALS (van den Berg et al., 2019). A promising and robust approach is the measurement of white matter (WM) degeneration by the use of diffusion tensor imaging (DTI) (Kassubek and Müller, 2020). In addition to the voxel-wise analysis of the whole brain (Müller et al., 2016), a tract-of-interest (TOI)-based approach can be used to analyze specific cerebral WM pathways that are involved in the progression of ALS (Kassubek et al., 2014). Longitudinally, the spread of pathology is reflected by tract-specific alterations in DTI metrics (Kassubek et al., 2018), which correlates with the clinical severity of the disease (Baldaranov et al., 2017).

Longitudinal MRI examinations of the brain are time-consuming, costly, and can be a burden for patients with ALS (especially in advanced disease stages). Thus, a careful design of such studies is mandatory. One of the most crucial variables in the conceptualization of a longitudinal study is the sample size as samples that are too small might lead to non-significant results of true effects (Blain et al., 2007; Alruwaili et al., 2019). Another essential aspect is the schedule of follow-up measurements, that is, the number of follow-ups and the time intervals between them. On one hand, it must be taken into consideration when the effect, that is, a change in diffusion metrics, can be measured at the earliest (Kalra et al., 2020), and on the other hand, the timing of follow-up measurements can be substantial for the validity of the results (Müller et al., 2021a). Confounding factors such as general and subject-specific noise cause diffusion metrics to be subject to measurement errors (Müller et al., 2013). A higher measurement error results in higher measurement uncertainty, that is, the measured value probably does not directly reflect the true value. Then, the measurement uncertainties affect the test-retest reliability of DTI metrics, that is, the ability to obtain similar values from different acquisitions of the same subject (Vollmar et al., 2010; Koller et al., 2021). The presence of high measurement errors can potentially bias the temporal association of variables in longitudinal studies (Saccenti et al., 2020). Especially in patients with burdening neurodegenerative diseases, more subject-specific measurement artifacts are to be expected compared to healthy subjects. All these aspects might be a reason why previously reported *post-hoc* effect sizes of longitudinal FA changes in patients with ALS were only limited (Kassubek et al., 2018). This indicates that DTI study protocols may be improved to increase the reliability of DTI metrics that might potentially serve as technical biomarkers in studies.

The objective of this study was to evaluate the effects of measurement uncertainty and scheduling of follow-up measurements on statistical power and sample size in a longitudinal study of patients with ALS. The approach is based on fractional anisotropy (FA) along the corticospinal tract (CST) which represents neuropathological ALS stage one (Kassubek et al., 2018) and is a robust and sensitive DTI-based parameter for disease progression (Kocar et al., 2021). This study aimed to establish a basis for the optimization of study protocols for longitudinal ALS imaging studies that are robust to different longitudinal FA decrease rates.

## Methods

### Participants

A total of 80 patients (58.5 ± 13.9 years, 48 male/32 female) with clinically definite or probable sporadic ALS according to the revised version of the El Escorial World Federation of Neurology criteria (Brooks et al., 2000) were included in the study. All patients underwent standardized clinical-neurological and routine laboratory examinations. None of the patients had any history of neurological or psychiatric disorders apart from ALS. The severity of disability as measured with the revised ALS functional rating scale (ALS-FRS-R) (Cedarbaum et al., 1999) was 40 ± 5 (range 23–48). For analysis at the group level, 50 age- and sex-matched healthy controls (54.3 ± 9.8 years, 32 male/18 female) were analyzed. For test-retest measurements, 14 healthy subjects (age 36.3 ± 11.2 years, 6 male/8 female) participated. All healthy controls had no history of any medical condition and were medication-free.

All patients and healthy controls gave written consent for the MRI protocol according to the institutional guidelines. The study was approved by the Ethical Committee of the University of Ulm, Germany (reference # 19/12), and written consent was obtained from each participant.

### Magnetic resonance imaging data acquisition and processing

Test-retest measurements of 14 healthy subjects were acquired on the same 1.5 T MRI scanner (Magnetom Symphony, Siemens Medical, Erlangen, Germany) with 151 ± 112 days in-between both scanning sessions. Since the reliability of diffusion metrics is affected by the number of GD (Teipel et al., 2011), each scanning session consisted of two DTI sequences with different protocols: Protocol A consisted of 52 gradient directions (GD) including four *b*0 directions (*b* = 1,000 s/mm^2^, voxel size 2.0 mm × 2.0 mm × 2.8 mm, 128 × 128 × 64 matrix, *TE* = 95 ms, *TR* = 8,000 ms) and protocol B consisted of 62 GD including two *b*0 directions (*b* = 1,000 s/mm^2^, voxel size 2.5 mm × 2.5 mm × 2.5 mm, 128 × 128 × 64 matrix, *TE* = 102 ms, *TR* = 8,700 ms). Between both sequences, the participants remained in the scanner. The ratio of the number of GD and *b*0 direction additionally influences the reliability of the diffusion metrics (Zhu et al., 2009). Optimization of the test-retest protocols was not performed in this respect, since the objective was not to minimize the error but to estimate the error from protocols commonly used in ALS studies (Kassubek et al., 2014; Müller et al., 2016; Behler et al., 2022; Münch et al., 2022).

The signal-to-noise ratio (SNR) may be lowered in patients with neurodegenerative diseases due to subject-related factors (Müller et al., 2013). Therefore, all 80 patients with ALS and a healthy control group (50 subjects) underwent protocol C at a 3.0 T MRI scanner (Allegra, Siemens Medical, Erlangen, Germany), as this has a higher SNR compared to a 1.5-T MRI scanner. Protocol C consisted of 49 GD including one *b*0 direction (*b* = 1,000 s/mm^2^, voxel size 2.2 mm × 2.2 mm × 2.2 mm, 96 × 128 × 52 matrix, *TE* = 85 ms, *TR* = 7,600 ms).

#### Diffusion tensor imaging analysis

For DTI data post-processing, the software *Tensor Imaging and Fiber Tracking* (TIFT) (Müller et al., 2007) was used. First, all DTI data sets were checked for eddy current distortions, underwent quality control (Müller et al., 2011), and were resampled to an isotropic 1 mm grid. This was followed by a non-linear spatial normalization to the Montreal Neurological Institute (MNI) stereotaxic standard space (Brett et al., 2002) by using study-specific DTI templates as described previously in detail (Müller et al., 2012). Baseline and follow-up DTI data sets of the test-retest group were aligned using a halfway linear registration (Menke et al., 2014) before MNI normalization. FA maps were calculated from each data set and, finally, smoothed with a Gaussian filter with an 8 mm full width-at-half-maximum.

#### Fiber tracking

An averaged data set of MNI transformed controls’ data was used for the identification of the CST by a seed-to-target TOI-based approach (Kassubek and Müller, 2020). A deterministic streamline fiber tracking approach (Müller et al., 2009) was used at which the FA threshold was set at 0.2 (Kunimatsu et al., 2004) and the Eigenvector scalar product threshold was set at 0.9. The seed regions had a radius of 5 mm and the target regions had a radius of 10 mm. In a final step, the technique of tract-wise fractional anisotropy statistics (TFAS) was applied to select FA values underlying the fiber tracks for arithmetic averaging. Bihemispheric mean FA values of fiber tracts were averaged and corrected for age (Behler et al., 2021). An age correction of the FA maps of the test-retest group was not performed since this group was only used to determine the measurement uncertainty which could be assumed to be independent of age and gender.

### Simulation of longitudinal trajectories

For the single subject *i*, the FA value *FA*_*t*,*i*_ of a follow-up measurement at time *t* after the baseline measurement (*t* = 0) can be calculated based on a linear relationship with a subject-specific rate of FA change β_*i*_:


(1) $$F\,A_{t,i}=F\,A_{0,i}+\beta_{i}\cdot t$$

However, FA values, like any other measured value, are not without measurement error. The measured FA value ${\hat{F\,A}}_{t,i}$ is composed of the real value and a measurement error ε_*t*_, which differs for each measurement:


(2) $${\hat{F\,A}}_{t,i}=F\,A_{t,i}+\varepsilon_{t}$$

**FIGURE 1:**
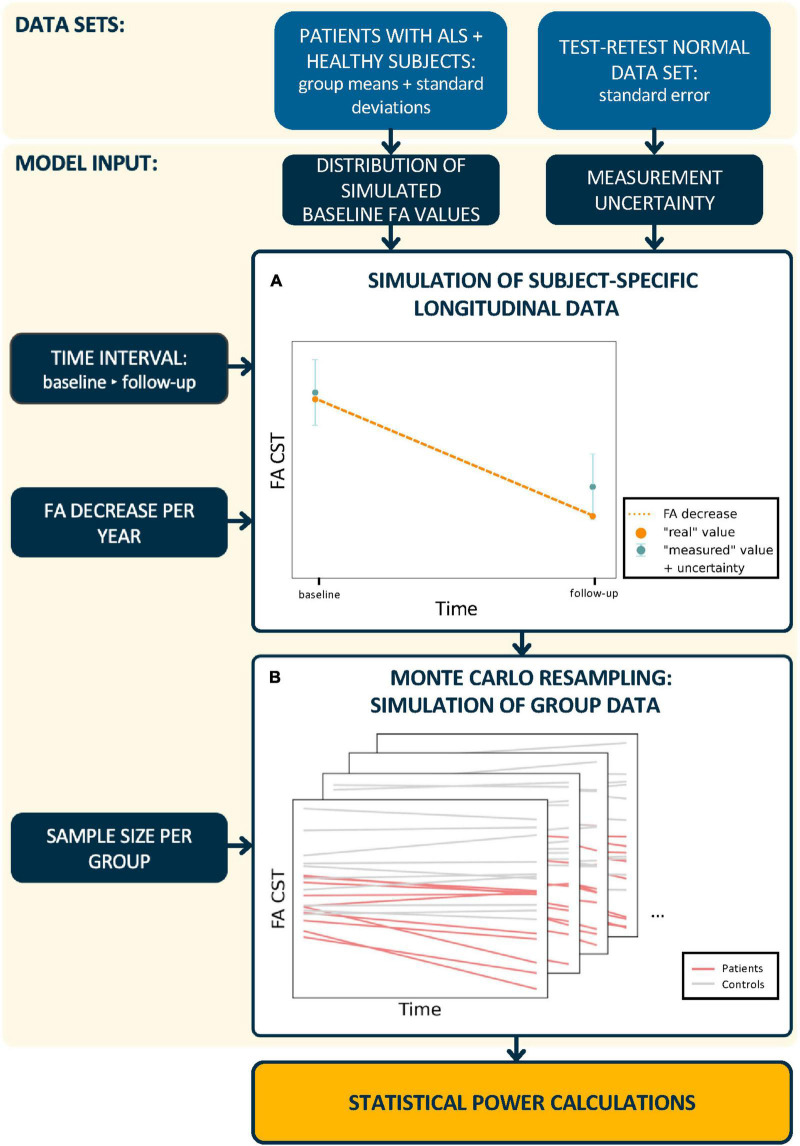
Schematic workflow of statistical power calculations. In a first step, **(A)** subject-specific longitudinal fractional anisotropy (FA) values in the corticospinal tract (CST) were simulated. Therefore, synthetic baseline values for patients and healthy controls were generated from real subject data distributions. The calculation of synthetic “measured” follow-up FA values incorporated a predefined FA decrease and measurement uncertainty. In a second step, **(B)** longitudinal trajectories were generated for *n* subjects per group and the statistical power was calculated from 2,000 Monte Carlo resampled data sets. This procedure was performed for different time intervals between baseline and follow-up sessions, measurement uncertainties, longitudinal FA decrease rates, and sample sizes.

For the simulation of longitudinal FA values (Figure 1A), this results in:


(3) $${\hat{F\,A}}_{t,i}={\hat{F\,A}}_{0,i}-\varepsilon_{0}+\beta_{i}\cdot t+\varepsilon_{t}$$

The measurement errors, that is, the measurement uncertainties, ε_0_ and ε_*t*_, of the baseline and the follow-up measurement at time *t* originate from a normal distribution (Table 1).

**TABLE 1:** Description of the distributions used for longitudinal group data simulations.

Variables generated in simulation	Distribution	Basis for distribution parameters
“Measured” baseline FA values (group- and subject-specific)	${\hat{F\,A}}_{0,i}∼𝒩\,\left(\mu_{\text{patients}},\sigma_{\text{patients}}\right)$	μ_patients_ and σ_patients_from 80 patients with ALS
	${\hat{F\,A}}_{0,i}∼𝒩\,\left(\mu_{\text{controls}},\sigma_{\text{controls}}\right)$	μ_controls_ and σ_controls_ from 50 healthy controls
Measurement uncertainty (measurement-specific)	ε_*i*_ ∼ 𝒩(0,*SEM*)	*SEM* from 14 test-retest normal data sets
Longitudinal FA decrease (subject-specific)	β_*i*_ ∼ 𝒩(μ_β_*i*__, σ_β_*i*__)	Specification of μ_β_*i*__ and σ_β_*i*__ based on previous studies (Cardenas-Blanco et al., 2016; Baldaranov et al., 2017; Kassubek et al., 2018; Kalra et al., 2020)

All random variables are normally distributed with mean μ and standard deviation σ.

To extend this approach to *n* subjects, a between-subject variability of the intercept ${\hat{F\,A}}_{0,i}$ and slope β_*i*_ is considered, that is, these are subject-specific Gaussian random effects (Table 1).

### Reliability analysis

To assess the measurement reliability of the FA values in the CST, the intraclass correlation coefficient (*ICC*) was calculated from the test-retest data sets of the healthy subjects with the following specifications: two-way mixed effect, single rater, that is, MRI scanner, and absolute agreement (Koo and Li, 2016). *ICC* values < 0.50 indicate poor reliability, values between 0.50–0.75 indicate moderate reliability, values between 0.75–0.90 indicate good reliability, and values > 0.90 indicate excellent reliability. The 95% confidence intervals (CI) were considered for this assessment.

The standard errors ε of a FA value (Table 1) were estimated using the standard error of the mean (*SEM)* from the *ICC* (Weir, 2005):


(4) $$S\,E\,M=\frac{T\,S\,S}{\left(k-1\right)}\cdot \sqrt{I\,C\,C\cdot \left(1-I\,C\,C\right)}$$

with *TSS* as the total “within-samples” sum of squares and *k* as the number of measurements.

### Statistical power calculations

The statistical power was evaluated for the comparison of longitudinal FA decreases between a group of patients and a group of healthy controls using a Monte Carlo simulation approach (Figure 1B) for different study designs. The simulations of FA values at the follow-up session were based on subject-specific longitudinal FA decrease rates only in patients with ALS since the annual longitudinal FA decrease for healthy controls was set to null. The FA decrease group mean in patients with ALS was specified based on measurements in previous studies:

- to 0.05% representing a group with a slow longitudinal FA decrease (Baldaranov et al., 2017).
- to 2.00% representing a group of patients with intermediate longitudinal FA decrease rates (Kassubek et al., 2018; Kalra et al., 2020).
- to 3.50% representing a group with a fast longitudinal FA decrease (Cardenas-Blanco et al., 2016).

It has to be noted, in general, however, that the speed of deterioration of a technical measurement like MRI/DTI not necessarily has to be associated with the speed of progression at the clinical level, because, in complex diseases like ALS, many (individual) factors may influence the clinical disease course. Since the simulation was based on subject-specific trajectories, the coefficient of variation of those mean FA decrease rates was set to 67% in patients with ALS.

The algorithm to calculate the statistical power for a given sample size per group *n* and a given effect size, that is, mean longitudinal FA decrease rate in the patients with ALS, at a significance level of 0.05 is as follows:

Step 1: The time interval between the baseline and the follow-up session, the number of scans per session *m*, and the magnitude of the measurement uncertainty were specified. The time intervals chosen between baseline and follow-up sessions varied from 30 to 180 days, which are typical intervals in longitudinal studies (Baldaranov et al., 2017; Kassubek et al., 2018; Kalra et al., 2020).

Step 2: Based on cross-sectional data estimated from studies with real subjects (Figure 1A), synthetic “measured” baseline FA values ${\hat{F\,A}}_{0,i}$ were generated for each subject from a normal distribution (Table 1) and *m* measurement repetitions were simulated by resampling using the normal distribution of the measurement uncertainty [equation (2)].

Step 3: A longitudinal FA decrease rate β_*i*_ was assigned to each subject (Table 1) and longitudinal FA values (*m* scans at one follow-up session *t* days after the baseline session) were calculated for each subject according to [equation (3)].

Step 4: The group comparison of longitudinal FA change was analyzed with a two-sided independent *t*-test and the *p*-value was calculated.

Step 5: Steps 2–4 were iterated 2,000 times and the number of significant iterations was obtained. Statistical power was estimated as the proportion of iterations with statistically significant results out of all iterations (Figure 1B).

## Results

### Simulation input

The test-retest reliability of the FA of the CST was determined for two different DTI protocols (1.5 T scanner) and ranged from good to excellent with an *ICC* of 0.91 [CI: (0.74, 0.97)] for protocol A and an *ICC* of 0.97 [CI: (0.91, 0.99)] for protocol B. According to equation (4), the standard error ε of a FA value was calculated to be 0.00134 for protocol A and 0.00054 for protocol B, respectively. The analysis showed that DTI protocols on the same scanner could lead to different magnitudes of measurement uncertainty of FA values in the CST. In the following, the standard error ε of protocol A is referred to as “high measurement uncertainty” and that of protocol B as “low measurement uncertainty,” since the latter provided more reliable values. As the magnitude of the measurement uncertainty directly affects the correlation structure of longitudinal data, the simulations and calculation of statistical power were performed for both measurement error magnitudes, that is, a low and high measurement uncertainty.

The tract-based group analysis of cross-sectional data showed a mean FA value of 0.326 (SEM, 0.002) with a standard deviation of 0.018 for the CST for patients with ALS and a mean FA value of 0.339 (SEM, 0.003) with a standard deviation of 0.023 for healthy controls.

### Monte Carlo statistical power estimate

Overall, for both measurement uncertainties, it was shown that multiple repeated scans per session led to an increase of the statistical power in detecting longitudinal changes in the FA in the CST under otherwise identical conditions, that is, time interval and group size.

**FIGURE 2:**
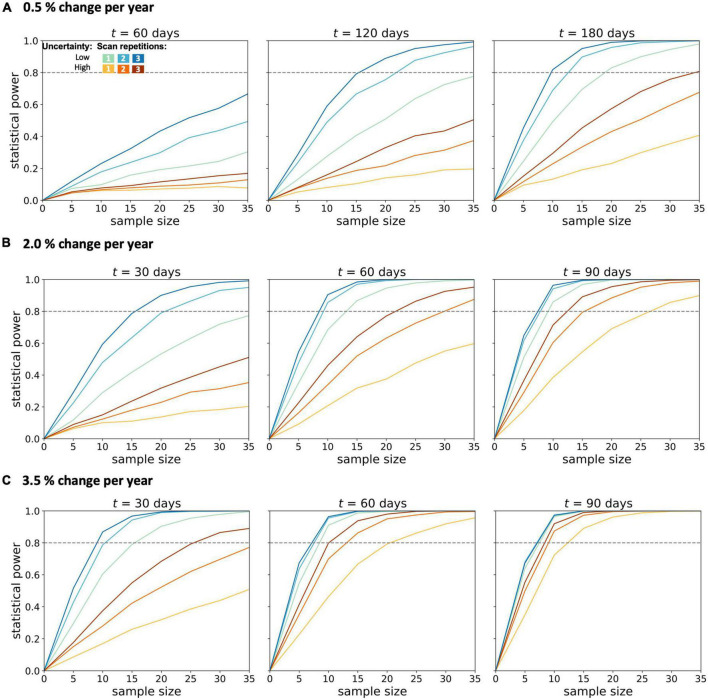
Statistical power for longitudinal diffusion tensor imaging studies in amyotrophic lateral sclerosis. Calculations were performed for **(A)** 0.5%, **(B)** 2.0%, and **(C)** 3.5% change per year of the fractional anisotropy in the corticospinal tract. Longitudinal simulations for a patient and a healthy control group were performed for different sample sizes per group, two magnitudes of measurement uncertainty, one to three scans per session, and time intervals *t* between baseline and the follow-up session.

From the simulation of a 0.5% longitudinal FA decrease in the CST per year (slow longitudinal FA decrease), it was shown at the analyzed time intervals of 60, 120, and 180 days (Figure 2A) that a second scan per session resulted in increased statistical power across both measurement uncertainties and all time intervals. Due to the lower change per year, the third scan per session led to a further increase in the statistical power which was similar to the increase due to a second scan for high measurement uncertainty.

**FIGURE 3:**
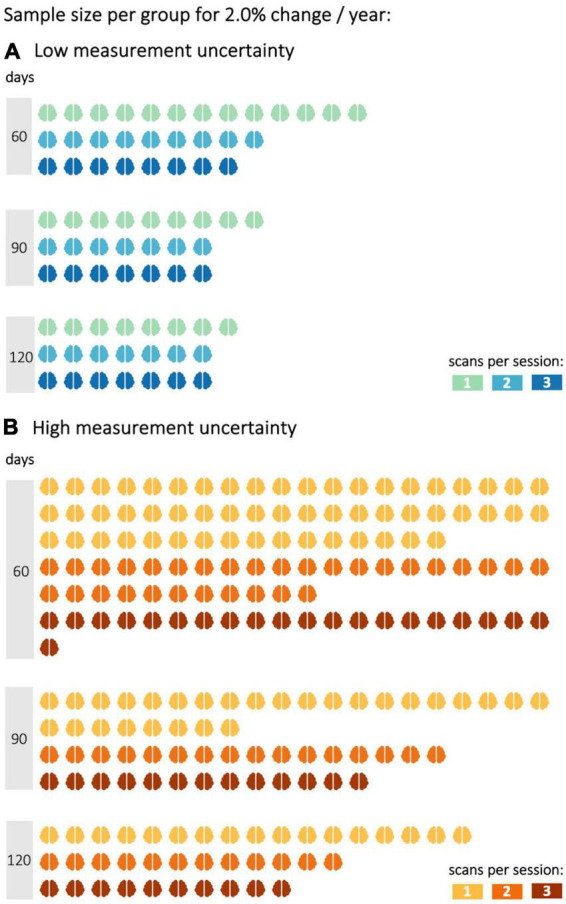
Sample size per group to reach a statistical power of 0.8 with different decrease rates in fractional anisotropy (FA). FA values were either subject to **(A)** low or **(B)** high measurement uncertainty. Statistical power calculations were performed for 60, 90, and 120 days between baseline and follow-up sessions and one to three scans per session.

In the simulations with 2.0–3.5% longitudinal FA decrease, shorter time intervals were also analyzed, since effects should be observable after shorter time intervals with a more pronounced longitudinal decrease. In the simulation with an average annual FA decrease of 2.0% (Figure 2B), the statistical power of 0.8 could not be achieved with either low or high measurement uncertainty for a time interval of 30 days and one scan per session for less than 35 subjects per group. The second scan increased the statistical power so that a statistical power of 0.8 could be reached with 20 subjects per group (with a given low measurement uncertainty). A subsequent, third scan per session led to further improvement of the statistical power for both measurement uncertainties. For measurements with low measurement uncertainty, the third scan did not further increase the statistical power. This positive effect of the third scan per session was lower the higher the time intervals between the baseline and the follow-up session were. Thus, with a 90-day interval between baseline and follow-up, the third measurement did not provide any additional advantage over a two-time repeated scan. The analysis of the sample sizes per group which was needed to reach an effect size of 0.8 showed that, for FA values with high measurement uncertainty, the second scan per session resulted in a reduction of the required sample size per group by about 30–45% (Figure 3).

In the simulation with a longitudinal FA decrease of 3.5% per year in the CST (“fast FA progressors”) (Figure 2C), it was shown that, with 90-days between baseline and follow-up measurement, a statistical power of more than 0.8 could already be achieved with 12 subjects per group, as well as for FA values with high measurement uncertainty. Repeated scans per session resulted in an increase of the statistical power in case of high measurement uncertainties and/or shorter time intervals (30 days). For a low measurement uncertainty, however, this improvement could not be demonstrated already at follow-up measurements 90-days after baseline. For measurements subject to high measurement uncertainty, the third scan brought no further advantage after 90-days, as compared to two scans per session.

## Discussion

This study investigated how the potential of the DTI-based metric FA as a non-invasive progression marker may be further optimized to monitor longitudinal changes in the FA during the disease progression of ALS. The influences of sample size, scheduling of baseline and follow-up sessions, and measurement uncertainty on the statistical power were assessed for longitudinal FA studies in the CST. Follow-up FA values were simulated for patients with ALS and healthy controls based on real baseline data distributions. Based on these synthetic longitudinal FA values, it could be demonstrated that a second scan at each session substantially increased the statistical power of such studies, especially for uncertain measurements with a limited SNR, for example, due to subject-related factors (Müller et al., 2013). The application of these results will strengthen the reliability of the FA values, in line with SNR improvement by signal-averaging during individual scans (Farrell et al., 2007; Seo et al., 2019). Vice versa, the increased statistical power of a DTI protocol means that lower sample sizes suffice to measure small effects and/or effects after a short time, respectively. With repeated scans per session, longitudinal FA changes in the CST could be detected already after short time intervals. This can be useful to identify even small alterations in cerebral WM pathways (Bede and Hardiman, 2018; Müller et al., 2021b) or potentially even small treatment effects if studied in a given therapeutic intervention. From longitudinal simulations of MRI correlates of fast disease progression, it was shown that DTI can detect alterations in the CST with satisfactory statistical power even after a short time. This provides the opportunity to use DTI to differentiate between ALS variants with different progression rates (Baek et al., 2020; Kalra et al., 2020).

As additional scans are a burden for patients with neurodegenerative diseases, reliability analyses are often performed on healthy participants. Patients with ALS might present with reduced or restricted mobility, leading to suboptimal positioning within the scanner and/or discomfort. Breathing difficulties further interfere with lying supine in the MRI scanner. This may result in a decreased SNR and, therefore, decreased reliability of diffusion metrics. Therefore, measurement uncertainty of FA values determined for healthy subjects in this study might underestimate those in patients with ALS. Thus, it may be concluded that repeated scans at one visit are beneficial especially at an advanced disease stage to achieve sufficient statistical power even with small sample sizes. Of course, repeated scans per session can also be an additional burden, especially for patients in later disease stages, and it may be assumed that repeated scans per session are possible at baseline but might be declined by the patient at later follow-ups. However, even then the repeated scans at baseline have a high value for the longitudinal analysis, since the data may be automatically weighted. Therefore, time intervals between multiple follow-ups no longer bias the results (Müller et al., 2021a). Since several uncertainties of measurements might occur in a clinical study, depending on patient condition including disease progression, one possible improvement could be to scan subjects two times during one visit.

A separate analysis of data from 1.5 to 3.0 T MRI acquisition protocols showed that similar FA values could be obtained in patients with ALS at different field strengths (Kassubek et al., 2014). Therefore, measurement uncertainties acquired from 1.5 T scanner data could be used together with group data obtained at a 3.0 T scanner: 3.0 T data show a higher SNR compared to 1.5 T data, thus 1.5 T data were only used for uncertainty estimation in this study and simulations at the group level were performed on data recorded on a 3.0 T scanner. The reliability analysis of two different 1.5 T scanner protocols showed that the *ICC* of the FA in the CST, obtained by a tract-based approach, was in the same order of magnitude as reported for a 3.0 T scanner (Lewis et al., 2020). This finding is not surprising, since the reliability of diffusion metrics is not affected by the field strength alone (Vollmar et al., 2010) but also by the number of GD (Teipel et al., 2011), DTI data processing pipelines (Thieleking et al., 2021), and the MRI scanner itself (Palacios et al., 2017). Although field strength is only one of the several factors affecting test-retest reliability, scanners with different field strengths in multicenter studies lead to increased inter-site variability in diffusion metrics. This has an additional negative impact on the statistical power of longitudinal studies due to larger variability between subjects from different sites. Since multicenter studies are often required in rare diseases such as ALS, this limitation can be addressed with robust harmonization methods that reduce inter-site variability while preserving biological variability (Pinto et al., 2020). An approach using linear correction for scanner effects in multicenter longitudinal studies showed better estimates accounting for the within-subject variability (Venkatraman et al., 2015); an analysis approach for harmonizing multicenter DTI data have been reported previously (Müller et al., 2016; Kalra et al., 2020). Since the measurement uncertainty may differ between sites, it could be assumed that the acquisition of multiple scans per visit also might have a positive effect on the harmonization of multicenter studies and their evaluations.

This study is not without limitations. The sample size of the test-retest cohort was limited, and these DTI data sets were acquired on a different MRI scanner than those of the groups providing basic information for longitudinal simulation. To strengthen the power of such simulations, test-retest measurements on the scanner of the planned study would be ideal. This study focused on longitudinal alterations in FA in the CST because the CST alterations are to be robustly found early in the disease process of ALS (Menke et al., 2017; Bede and Hardiman, 2018; Kassubek et al., 2018; Baek et al., 2020). The reliability of other tract systems might be different from those of the CST (Marenco et al., 2006; Luque Laguna et al., 2020), leading to a limited transferability of results for optimal time intervals and sample sizes to other WM pathways. For the simulation, the same magnitude of time-independent measurement uncertainty was used for patients and healthy subjects, but this might not completely represent reality, since it can be assumed that the quality of the patient data sets might be worse than that of the healthy subjects and thus, the measurement uncertainty would be higher for patients. Also, increasing disease severity during the course could further affect the quality and in that way the measurement uncertainty.

In summary, this study demonstrated that the statistical power of longitudinal DTI studies in ALS can be substantially increased by multiple scans of the same subject per session, especially in limited sample sizes. Such optimized study protocols can help to establish FA as an imaging biomarker in ALS, especially to monitor disease progression not only in the natural history but also under future disease-modifying therapeutic approaches.

## Data availability statement

The raw data supporting the conclusions of this article will be made available by the authors, without undue reservation.

## Ethics statement

The studies involving human participants were reviewed and approved by Ethical Committee of the University of Ulm, Germany. The patients/participants provided their written informed consent to participate in this study.

## Author contributions

AB: study design, data analyis, simulations and interpretation of data, and drafting of the manuscript. DL and AL: interpretation of data and critical revision of the manuscript for intellectual content. H-PM and JK: study concept and design, interpretation of data, and critical revision of the manuscript for intellectual content. All authors contributed to the article and approved the submitted version.
